# Comprehensively benchmarking applications for detecting copy number variation

**DOI:** 10.1371/journal.pcbi.1007069

**Published:** 2019-05-28

**Authors:** Le Zhang, Wanyu Bai, Na Yuan, Zhenglin Du

**Affiliations:** 1 College of Computer Science, Sichuan University, Chengdu, China; 2 Medical Big Data Center, Sichuan University, Chengdu, China; 3 Zdmedical, Information polytron Technologies Inc. Chongqing, Chongqing, China; 4 BIG Data Center, Beijing Institute of Genomics, Chinese Academy of Sciences, Beijing, PR China; Ottawa University, CANADA

## Abstract

**Motivation:** Recently, copy number variation (CNV) has gained considerable interest as a type of genomic variation that plays an important role in complex phenotypes and disease susceptibility. Since a number of CNV detection methods have recently been developed, it is necessary to help investigators choose suitable methods for CNV detection depending on their objectives. For this reason, this study compared ten commonly used CNV detection applications, including CNVnator, ReadDepth, RDXplorer, LUMPY and Control-FREEC, benchmarking the applications by sensitivity, specificity and computational demands. Taking the DGV gold standard variants as a standard dataset, we evaluated the ten applications with real sequencing data at sequencing depths from 5X to 50X. Among the ten methods benchmarked, LUMPY performs the best for both high sensitivity and specificity at each sequencing depth. For the purpose of high specificity, Canvas is also a good choice. If high sensitivity is preferred, CNVnator and RDXplorer are better choices. Additionally, CNVnator and GROM-RD perform well for low-depth sequencing data. Our results provide a comprehensive performance evaluation for these selected CNV detection methods and facilitate future development and improvement in CNV prediction methods.

## Author summary

As an important type of genomic structural variation, CNVs are associated with complex phenotypes because they change the number of copies of genes in cells, affecting coding sequences and playing an important role in the susceptibility or resistance to human diseases. To identify CNVs, several experimental methods have been developed, but their resolution is very low, and the detection of short CNVs presents a bottleneck. In recent years, the advancement of high-throughput sequencing techniques has made it possible to precisely detect CNVs, especially short ones. Many CNV detection applications were developed based on the availability of high-throughput sequencing data. Due to different CNV detection algorithms, the CNVs identified by different applications vary greatly. Therefore, it is necessary to help investigators choose suitable applications for CNV detection depending upon their objectives. For this reason, we not only compared ten commonly used CNV detection applications but also benchmarked the applications by sensitivity, specificity and computational demands. Our results show that the sequencing depth can strongly affect CNV detection. Among the ten applications benchmarked, LUMPY performs best for both high sensitivity and specificity for each sequencing depth. We also give recommended applications for specific purposes, for example, CNVnator and RDXplorer for high sensitivity and CNVnator and GROM-RD for low-depth sequencing data.

This is a *PLOS Computational Biology* Benchmarking paper.

## Introduction

Copy number variation (CNV) is a type of genomic structural variation that contains segmental duplications or deletions of a DNA fragment; the CNV size usually ranges from 1 kb to 3 Mb[1]. CNVs are found widely in individual human genomes, and they seldomly lead to genetic diseases[2]. CNVs can change the number of copies of a gene present in cells, thus affecting the coding sequences of genes, and they are associated with complex phenotypes [3]. CNVs also play an important role in the susceptibility or resistance to human diseases, such as cancer [4], Alzheimer disease [5], autism [6] and psoriasis [7].

Previously, researchers developed several experimental methods to explore CNVs, such as fluorescence in situ hybridization (FISH) and array comparative genomic hybridization (aCGH) [8], but the low resolution of these methods (approximately 5~10 Mbp for FISH and 10~25 kbp for aCGH) [9] presents a bottleneck for the detection of short CNVs [10]. In the last decade, Next Generation Sequencing (NGS) technology has enabled precise detection of CNVs, making it possible to identify small variants as short as 50 bp[11]. Many CNV detection algorithms were developed by NGS platforms.

The Read Depth (RD, or Read Count (RC))[12] and Pair-End Mapping (PEM, or Read Pair (RP))[13] algorithms are the most popular methods for CNV detection, and they use statistical models and clustering approaches for CNV detection[14], respectively. RD-based methods are good at detecting exact copy numbers, large insertions and CNVs in complex genomic region classes, whereas PEM-based methods can efficiently not only identify insertions and deletions but also locate mobile element insertions, inversions, and tandem duplications[14].

Many CNV detection methods have been developed based on the RD or PEM algorithms (Table 1). CNVnator is based on a statistical MSB model. It provides not only high sensitivity (86–96%) and genotyping accuracy (93–95%) but also a low false-discovery rate (3–20%)[15]. ReadDepth is based on a statistical CBS model, and it can interpret overdispersed data for better breakpoint estimation[16]. Control-FREEC is one of the most widely used RD-based CNV detection software programs, and it uses matched case-control samples or GC content to correct copy number[17]. CNVrd2 computes segmentation scores by integrating the linear regression algorithm[18] into a Bayesian normal mixed model; thus, it has the highest paralog ratio[19]. cn.MOPS decomposes variations in the depth of coverage across multiple samples into integer copy numbers and noise by means of its mixture components and Poisson distributions[20]. RDXplorer is based on the Event-Wise Testing (EWT) algorithm, which is a method based on significance testing, and the median size of detected CNVs is much longer than that using PEM methods[9]. Canvas is a favored tool for both somatic and germline CNV detection in large-scale sequencing studies, and it implements all steps of the variant calling workflow[21]. GROM-RD is a control-free CNV algorithm combining excessive coverage masking, GC bias mean and variance normalization[22]. iCopyDAV is a modular-framework based on DoC approaches[23]. RSICNV detects CNVs using the robust segment identification algorithm with negative binomial transformations[24]. LUMPY integrates the CNV detection methods of RD and PEM and allows for more sensitive CNV discovery[25].

**Table 1:** CNV detection methods on WGS data.

Software	Methods	Algorithm detail	Input data	Publish	Latest update	Accessibility	URL	Programing Language	#Citations
^#^Canvas	RD	Expectation-maximization (EM) clustering	BAM	2011	2018/3	Y	https://github.com/Illumina/canvas	C#	29
^#^cn.MOPS	RD	Mixture Poisson model	BAM	2012	2018/10	Y	http://www.bioinf.jku.at/software/cnmops/cnmops.html	R	226
CNVeM	RD	Expectation-maximization (EM) algorithm	CSV	2013	NA	Y	https://omictools.com/cnvem-tool	C	14
CNVer	RP	Maximum-likelihood, Graphic flow	BAM	2010	2011/5	N	NA	C	158
^#^CNVnator	RD	Mean shift algorithm	BAM	2011	2016/11	Y	https://github.com/abyzovlab/CNVnator	C++	640
CNVrd2	RD	Expectation-maximization (EM) algorithm	BAM/SAM	2014	2015/11	Y	https://bioconductor.org/packages/release/bioc/html/CNVrd2.html	R	13
^#^Control-FREEC	RD	LASSO regression	BAM/SAM	2011	2018/8	Y	http://boevalab.com/FREEC/	C++	190
^#^GROM-RD	RD	Quantile normalization	BAM	2015	2017/5	Y	http://grigoriev.rutgers.edu/software/	C	7
^#^iCopyDAV	RD	DoC approaches	BAM	2018	2018/3	Y	https://github.com/vogetihrsh/icopydav	R,C++	1
JointSLM	RD	Population-based approach	SAM/BAM	2011	NA	N	NA	R	49
^#^LUMPY	RD, PEM	A probabilistic framework	BAM/CRAM	2014	2016/3	Y	https://github.com/arq5x/lumpy-sv	C++	157
mrCaNaVAR	RD	mrFAST	SAM	2009	2013/9	Y	http://mrcanavar.sourceforge.net/	C	685
^#^RDXplorer	RD	Event-wise testing algorithm	BAM	2009	2013/4	Y	https://sourceforge.net/projects/rdxplorer/	Python	496
^#^ReadDepth	RD	Circular binary segmentation algorithm	Bed Files	2011	2014/8	Y	https://github.com/chrisamiller/readDepth	R	150
^#^RSICNV	RD	Negative binomial transformations	BAM	2017	2017/7	Y	https://github.com/yhwu/rsicnv	C++	2

Note:

^#^ indicates the software used in this study.

Previous studies have surveyed CNV detection software with regards to specificity, sensitivity and computational demands, and they have evaluated their advantages and shortcomings. For example, Fatima et al. evaluate CNV detection software based on analysis of whole-exome sequencing (WES) data[26], and Junbo et al. evaluate six RD-based CNV detection software programs based on analysis of whole genome sequencing (WGS) data[27]. However, previous studies neither consider the impact of varied sequencing depth on the software performance nor use a standardized CNV dataset for evaluation based on analysis of real sequencing data. Our study not only adds several newer, untested software programs such as RSICNV, iCopyDAV and GROM-RD but also uses Database of Genomic Variants (DGV) as the gold standard so that our test results are more extensive and reliable[28]. Here, we surveyed ten frequently used methods of CNV detection in WGS data (Table 1), including CNVnator, ReadDepth, RDXplorer, LUMPY and Control-FREEC, and evaluated not only the detected CNV number, length distribution and result coincidence between different CNV methods but also the accuracy, sensitivity and computational demand under the conditions of different sequencing depths. Our study also compares the advantages and shortcomings of such CNV detection methods, providing useful information for researchers to select a suitable method.

## Materials and methods

### Study data

The sequencing data (94x) of the individual NA12878 were downloaded from the website of the 1000 Genomes Project[29] as evaluation data to compare the performance of CNV detection methods using real sequencing data. The DGV Gold Standard Variants for NA12878 were download from the Database of Genomic Variants (DGV)[28], and a previously published SV benchmark of NA12878[30] was also fetched from the FTP site (ftp://ftp.1000genomes.ebi.ac.uk/vol1/ftp/phase3/data/NA12878/)[31].

### Identification of CNVs in NA12878

After removing sequencing adapters and trimming consecutive low-quality bases from both the 5' and 3' end of the reads using an in-house Perl script, clean reads were subsampled by the sequencing depth of 5x, 20x, 10x, 30x, 40x and 50x using seqtk (https://github.com/lh3/seqtk) [32]. Then, the six datasets were mapped to the human reference genome (hg19) using BWA (V0.7.12) (http://bio-bwa.sourceforge.net/) [33] with default parameters. The Picard program (https://broadinstitute.github.io/picard/) [34] was used to sort mapping results to the BAM format. For CNV identification of NA12878, ten methods were used with default or recommended parameters, including CNVnator, ReadDepth, RDXplorer, LUMPY and Control-FREEC. The CNVs with lengths of more than 1 kb were kept as detected CNVs. The main parameters for each software program used are listed in S1 Table.

### Performance evaluation criteria

In the two datasets of the DGV Gold Standard Variants and the SV benchmark, the CNVs longer than 1 kb were merged by location overlap of more than 50% and were taken as the standard CNV dataset for performance evaluation (S1 Table). The identified CNVs of each method were regarded as true positive results if there was more than 50% overlap on chromosome locations compared with the standard CNV dataset; otherwise, they were regarded as true negative CNVs. Then, the true positive rates (TPRs) and the false discovery rates (FDRs) were calculated and compared. The formulas to calculate TPR and FDR are shown in Table 2. For computing time estimation, each application was run five times, and the average running times were recorded for the related standard deviation computation. To compare the memory usage of the applications, each application was run five times, and the average memory sizes were recorded for the related standard deviation[35–38] computation. The process used for performance evaluation is shown in S1 Fig.

**Table 2:** Formula to calculate TPR and FDR.

Measure	Formula	Illustration
TPR	$TPR\;=\;\frac{TP}{P}$	TP: the number of true positivis P:the number of positives in DGV
FDR	$FDR\;=\;\frac{FP}{TP+FP}$	TP: the number of true positives FP: the number of false positives

## Results

### Comparison of identified CNVs

**Fig 1:**
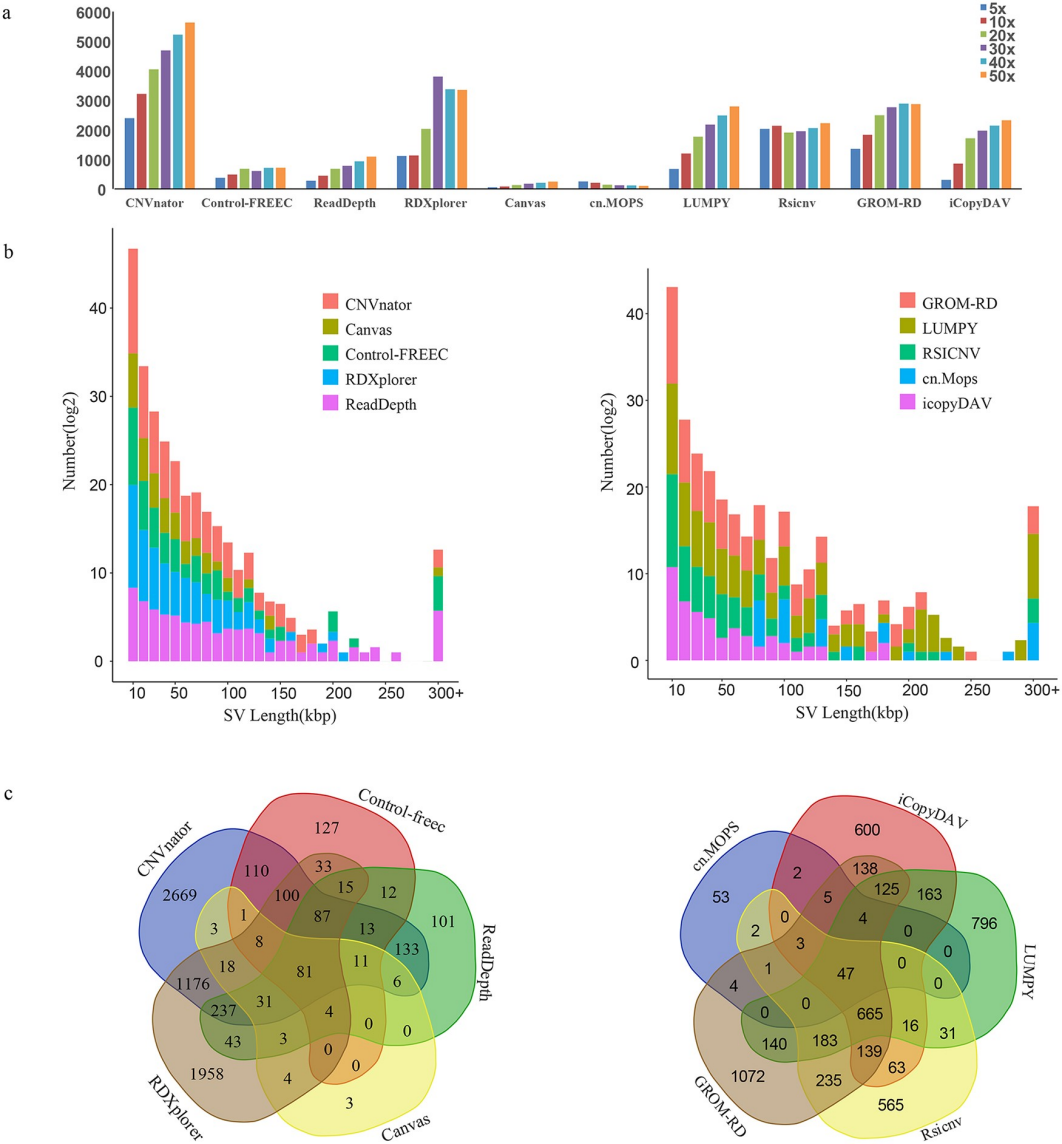
Statistics of the detected CNVs. (a) Detected CNV number. (b) Distribution of CNV size. (c) The Venn diagram of CNV detection methods.

With sequencing data with depths from 5X to 50X, ten methods were used to identify CNVs in NA12878 (shown in Table 1), and the tested CNVs were listed in the supplementary files(S1–S11 Files). As shown in Fig 1a, due to different CNV detection algorithms, the numbers of detected CNVs varied greatly. CNVnator and RDXplorer identified the most CNVs, whereas Canvas and cn.MOPS identified the fewest. In most cases, the number of CNVs identified were positively correlated with the sequencing depth. However, RDXplorer detected the most SVs at 30X depth, probably because the method was tested and optimized at a 30X sequencing depth[9].

On the other hand, each software program tended to detect CNVs of different sizes, ranging from less than 1 kb to several hundred kbp. As shown in Fig 1b, most methods identified many small CNVs shorter than 10 kb, whereas LUMPY and ReadDepth predicted more CNVs longer than 200 kb.

The detected CNVs for each method at a 30X sequencing depth were also compared in Fig 1c. Generally, CNVs identified by more than one method are more specific than those called by only one method[39]. As shown in Fig 1c, 98.27% of CNVs identified by Canvas were also identified by four other methods; the program with the next highest level of consistency with other methods was ReadDepth (87.00%), whereas CNVnator and RDXplorer identified the most CNVs that were only called in a single method.

### Sensitivity and specificity of CNV prediction

**Fig 2:**
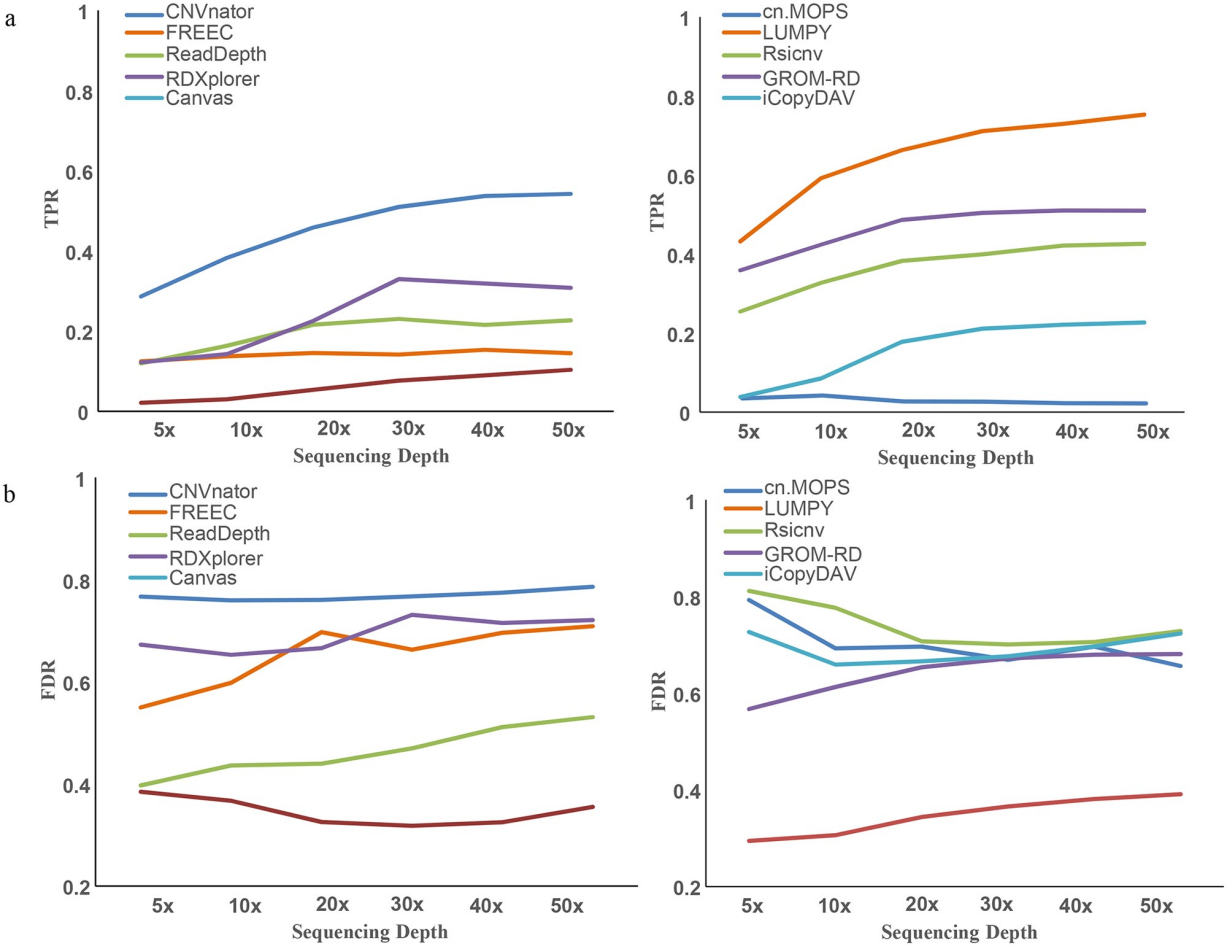
The evaluation of sensitivity and specificity of CNV detection methods. (a) TPR curves of the ten applications at sequencing depths from 5X to 50X. (b) FDR curves of the ten applications at sequencing depths from 5X to 50X.

As shown in Fig 2a, the TPR curves of the ten methods were plotted at six sequencing depths from 5X to 50X. At a low sequencing depth of 5X, the TPR of LUMPY reached 0.432, followed by CNVnator (0.370) and GROM-RD (0.359), which was much greater than other methods (0.021 to 0.254), implying that these three methods have greater sensitivity at low sequencing depth. At high sequencing depths of 30X and 50X, CNVnator also showed the highest TPR of 0.725 and 0.800, followed by LUMPY (0.711, 0.753) and RDXplorer (0.678, 0.621), implying higher sensitivity than other methods. Overall, at each sequencing depth from low to high, CNVnator and LUMPY had the best performance with respect to the sensitivity of CNV detection.

At increasing sequencing depths, the trends of the TPR curves were different from one another. For CNVnator, LUMPY and ReadDepth, the range with varying TPR was much wider (Fig 2a), and the TPR curve visibly increased, which indicates that the sensitivity of CNV detection is positively correlated with the sequencing depth. The TPR curve of RDXplorer also significantly increased with sequencing depth from 5X to 30X but reached a plateau at a 30X depth. This may result from the algorithm design as mentioned above.

Considering the sensitivity of detecting CNVs and sequencing costs, a sequencing depth of 30X provides the best value for CNV detection, as is indicated by the trends in the TPR curves (Fig 2a). However, the TPR curves were independent from sequencing depth for FREEC, cn.MOPS and Canvas (Fig 2a). With regards to the specificity of CNV detection methods, the FDR curves of Canvas and LUMPY were lower than the others, implying that the specificities of these two methods are better than those of the other methods, i.e., they predicted the least false positive results (Fig 2b). The FDR value of iCopyDAV reached a peak value at a 30X depth (0.878), followed by CNVnator (0.767) and RDXplorer (0.731), but these three methods also predicted the most CNVs (Fig 2b).

#### Computational demands

**Fig 3:**
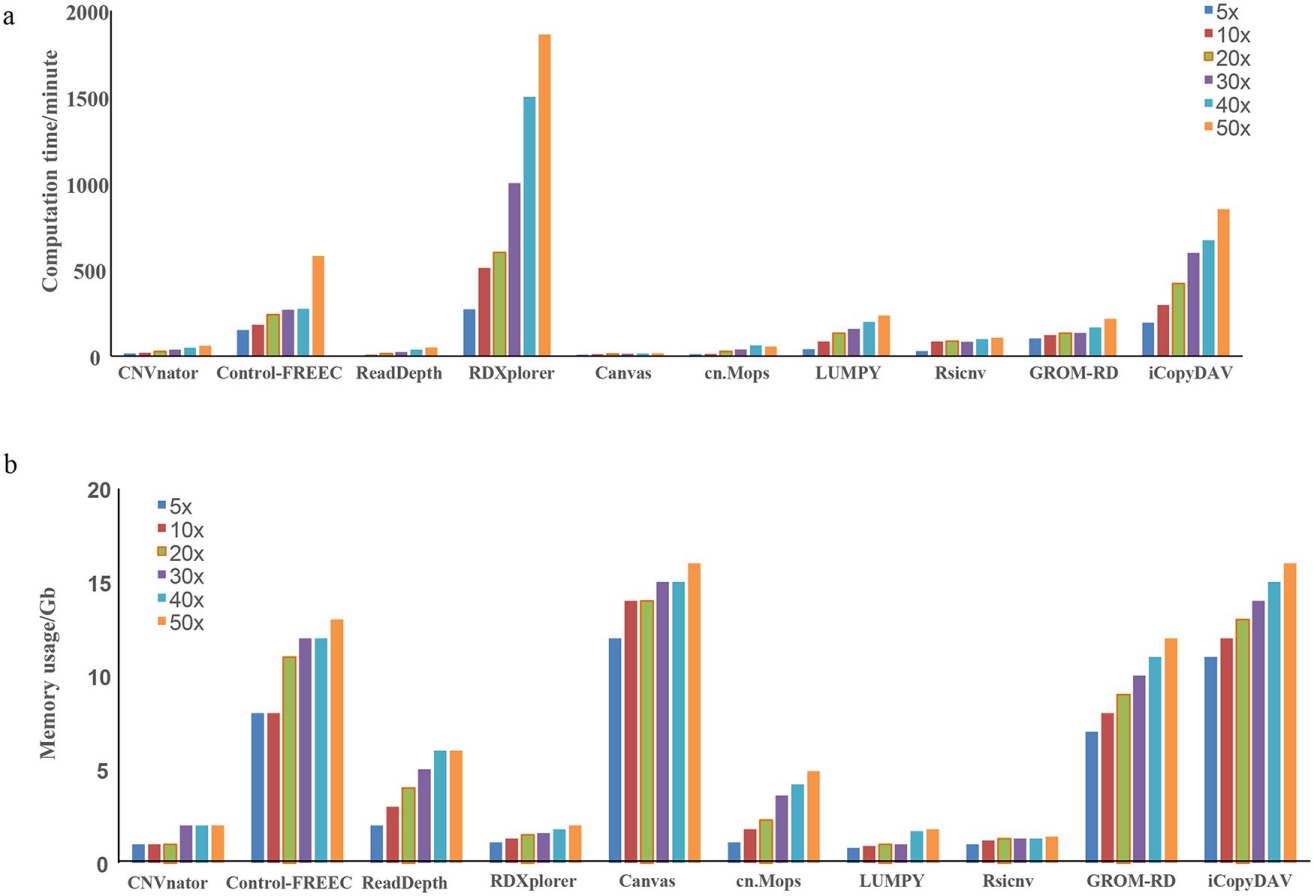
The computational demands of the ten methods. (a) Computation time as a function of sequencing depth from 5X to 50X. (b) Memory usage as a function of sequencing depth from 5X to 50X.

The computational demands of these methods with respect to computing time and memory usage are shown in Fig 3. Computing times of these ten applications increased with the increment of sequencing depth (Fig 3a). In particular, RDXplorer had the highest cost, followed by iCopyDAV and FREEC, with the other methods being comparable with low runtime costs. As shown in Fig 3b, the memory usage rates of these ten methods were positively related to sequencing depth. CNVnator, RDXplorer, RSICNV and LUMPY used the least amount of memory, while iCopyDAV, Canvas and FREEC needed more memory to run.

## Discussion

This study surveyed the performance of ten CNV detection applications with regards to sensitivity, specificity and computational demands over a range of sequencing depths.

We found that most CNVs detected by Canvas and ReadDepth could be explored by other methods, but CNVnator and RDXplorer identified many specific CNVs (Fig 1c). Of all the CNV detection methods, LUMPY showed the best performance in terms of both sensitivity and specificity, probably because LUMPY integrates two different algorithms of PEM and RD for CNV prediction[25], and the PEM algorithm can provide better mapping accuracy on highly repetitive genomic regions than RD-based methods in some cases.

Since TPR values for most methods were below 0.8 and the FDR values for most methods were above 0.3 (Fig 2), we believe that the sensitivity and specificity for CNV detection are not likely to be improved in the future.

Limiting the CNV detection algorithms studied, our results are consistent with a previous report[39]. For all the ten methods, including RD-based algorithms, the read depth distribution is affected by the following three major causes. First, the GC-content in genomes leads to PCR bias during the construction of sequencing libraries, and the genome regions with ultrahigh or ultralow GC-contents are difficult to sequence, so the read depths on these regions are uneven. Second, because the genome sequencing was performed using short reads and it is difficult to correctly map short reads to genome regions with highly repetitive sequences, false positive CNV results arise in most studies. Lastly, the valuation results for cn.MOPS fall short of expectations. Since the cn.MOPS method was designed for input data from multisamples, the sensitivity and specificity are both very low when inputting single samples.

The high FDR of CNV detection was also likely caused by the imperfectness of the standard CNV dataset. We also conducted the evaluation with another set of gold standard CNVs used in a previous study[40], but the evaluation results were similar. A possible explanation is that it is difficult to identify all the CNVs on real experimental data, in spite of the fact that many platforms were used to confirm the detected CNVs on DGV Gold Variations. Therefore, the standard CNV dataset may not comprise all the true CNVs in NA12878, and it may include some incorrect CNVs. For example, of all the CNVs in the standard CNV data set, 623 CNVs were not detected by any of the ten methods; these are most likely false positive detection results.

The benchmarking above was based on single subsampling on each sequencing depth. To avoid subsampling bias, we evaluated the effect of subsampling on CNV prediction using multiple random subsampling. As shown in S2 Fig, we calculated TPR and FDR using five times subsampling for each CNV program on 30X depth (S2a & S2b Fig), which is a typical depth for whole genome resequencing studies, and also subsampled five times on each depth for one program LUMPY (S2c & S2d Fig). Most CNV prediction results of multiple subsampling are steady and the trends of TPR and FDR curves of each program were consistent with previous benchmarking conclusions (Fig 2a & 2b).

The aim of this survey is to help researchers choose appropriate CNV detection methods according to their specific purposes and the features of their data. We suggest that (1) when low FDR is preferable, LUMPY and Canvas are better choices (Fig 2); (2) when high sensitivity is preferable, LUMPY, CNVnator and RDXplorer are better choices (Fig 2); and (3) if the speed/computation demand is the first priority, CNVnator and ReadDepth should be considered (Fig 3).

In this study, we employed the default or recommended parameters of each application for performance comparison. We plan to compare the best performance for each application by fine tuning the parameters and to include more recently published CNV applications in the future. Considering the limitations of sequencing data comprised of short reads, we are also preparing to evaluate CNV detection methods using long sequencing reads, such as PacBio or Oxford Nanopore, which may further improve the CNV prediction performance with regards to sensitivity and specificity.

## Supporting information

S1 Fig The evaluation workflow. (TIF)

S2 Fig Evaluation of sensitivity and specificity of CNV detection methods using five times subsampling. (a) TPR of the ten application at 30X depth using five times subsampling. (b) FDR of the ten application at 30X depth using five times subsampling. (c) TPR of Lumpy from 5X to 50X depth using five times subsampling at each depth. (d) FDR of Lumpy from 5X to 50X depth using five times subsampling at each depth.
(TIF)

S1 Table The detailed information concerning the tested software. (DOCX)

S1 File Standard CNVs for NA12878. (XLSX)

S2 File Detected CNVs using Canvas. (XLSX)

S3 File Detected CNVs using cn.MOPS. (XLSX)

S4 File Detected CNVs using CNVnator. (XLSX)

S5 File Detected CNVs using iCopyDAV. (XLSX)

S6 File Detected CNVs using GROM-RD. (XLSX)

S7 File Detected CNVs using Rsicnv. (XLSX)

S8 File Detected CNVs using Control-FREEC. (XLSX)

S9 File Detected CNVs using RDXplorer. (XLSX)

S10 File Detected CNVs using ReadDepth. (XLSX)

S11 File Detected CNVs using LUMPY. (XLSX)
