# Correction: Comprehensively benchmarking applications for detecting copy number variation

**DOI:** 10.1371/journal.pcbi.1007367

**Published:** 2019-09-20

**Authors:** Le Zhang, Wanyu Bai, Na Yuan, Zhenglin Du

Fig 2 is incorrect. The colours of the key and the graph did not match. The authors have provided a corrected version here.

**Fig 2:**
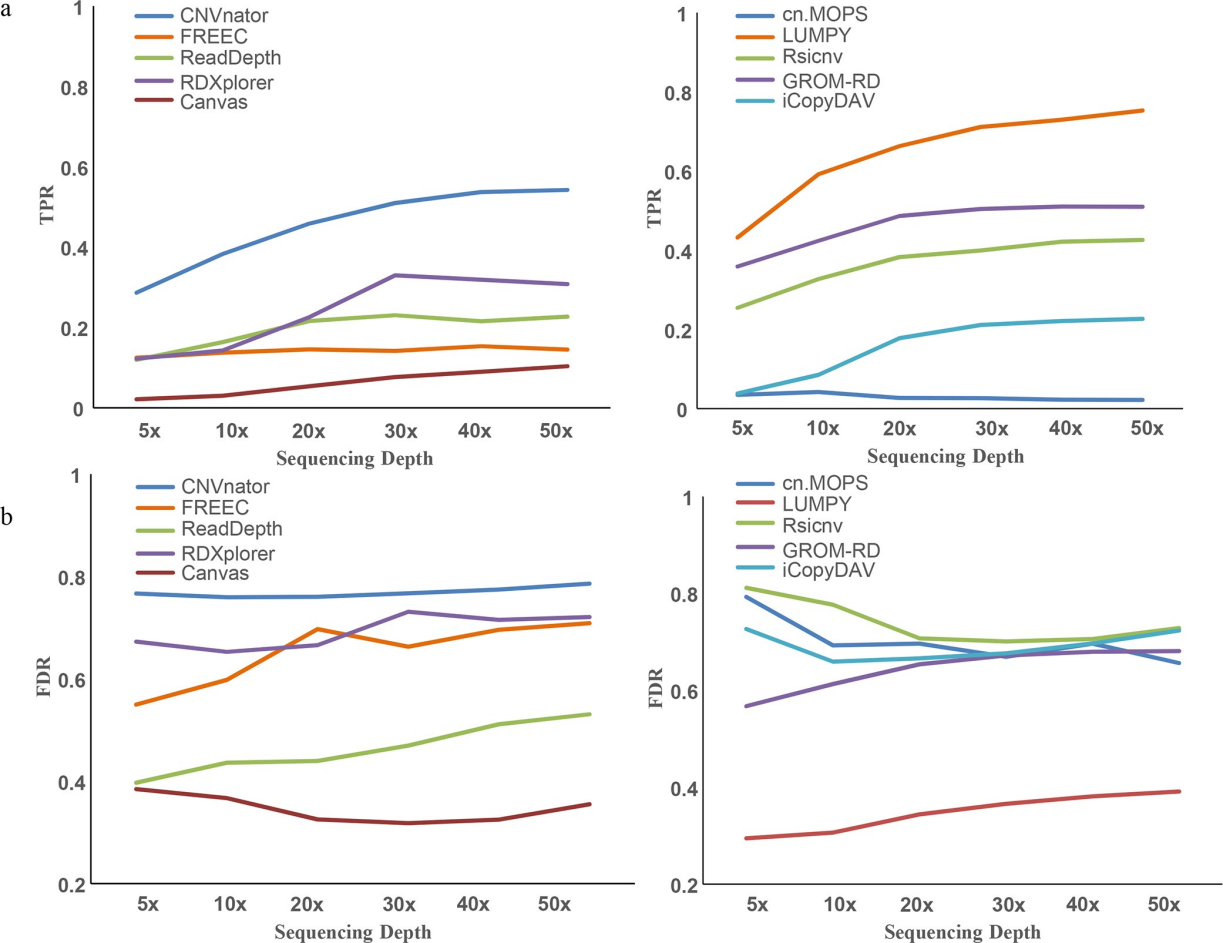
The evaluation of sensitivity and specificity of CNV detection methods. (a) TPR curves of the ten applications at sequencing depths from 5X to 50X. (b) FDR curves of the ten applications at sequencing depths from 5X to 50X.

Some sentences in the manuscript are incorrect:

In the **Abstract**:

‘If high sensitivity is preferred, CNVnator and RDXplorer are better choices.’

Should be

‘If high sensitivity is preferred, CNVnator and GROM-RD are better choices.’

In **Results: Sensitivity and Specificity of CNV prediction**–first paragraph:

‘At a low sequencing depth of 5X, the TPR of LUMPY reached 0.432, followed by CNVnator (0.370) and GROM-RD (0.359), which was much greater than other methods (0.021 to 0.254), implying that these three methods have greater sensitivity at low sequencing depth. At high sequencing depths of 30X and 50X, CNVnator also showed the highest TPR of 0.725 and 0.800, followed by LUMPY (0.711, 0.753) and RDXplorer (0.678, 0.621), implying higher sensitivity than other methods. Overall, at each sequencing depth from low to high, CNVnator and LUMPY had the best performance with respect to the sensitivity of CNV detection.’

Should be

‘At a low sequencing depth of 5X, the TPR of LUMPY reached 0.432, followed by GROM-RD (0.359) and CNVnator (0.287), which was much greater than other methods (0.021 to 0.254), implying that these three methods have greater sensitivity at low sequencing depth. At high sequencing depths of 30X and 50X, LUMPY also showed the highest TPR of 0.711 and 0.753, followed by CNVnator (0.510 and 0.542) and GROM-RD (0.504, 0.510), implying higher sensitivity than other methods. Overall, at each sequencing depth from low to high, LUMPY, CNVnator and GROM-RD had the best performance with respect to the sensitivity of CNV detection.’

In **Results: Sensitivity and Specificity of CNV prediction**–third paragraph:

‘The FDR value of iCopyDAV reached a peak value at a 30X depth (0.878), followed by CNVnator (0.767) and RDXplorer (0.731), but these three methods also predicted the most CNVs (Fig 2B).’

Should be

‘The FDR value of Rsicnv reached a peak value at a 5X depth (0.812), followed by cn.MOPS (0.793) and CNVnator (0.767), but CNVnator also predicted the most CNVs (Fig 2B).’

In the **Discussion–**third paragraph**:**

‘Since TPR values for most methods were below 0.8 and the FDR values for most methods were above 0.3 (Fig 2), we believe that the sensitivity and specificity for CNV detection are not likely to be improved in the future.’

Should be

‘Since TPR values for most methods were below 0.8 and the FDR values for most methods were above 0.3 (Fig 2), we believe that the sensitivity and specificity for CNV detection are likely to be improved in the future.’

In the **Discussion–**seventh paragraph**:**

‘We suggest that (1) when low FDR is preferable, LUMPY and Canvas are better choices (Fig 2); (2) when high sensitivity is preferable, LUMPY, CNVnator and RDXplorer are better choices (Fig 2); and (3) if the speed/computation demand is the first priority, CNVnator and ReadDepth should be considered (Fig 3).’

Should be

‘We suggest that (1) when low FDR is preferable, LUMPY and Canvas are better choices (Fig 2); (2) when high sensitivity is preferable, LUMPY, CNVnator and GROM-RD are better choices (Fig 2); and (3) if the speed/computation demand is the first priority, CNVnator and ReadDepth should be considered (Fig 3).’
