# Supplementary figures and images for: Comprehensively benchmarking applications for detecting copy number variation
Source: PLoS Comput Biol. 2019 May 28;15(5):e1007069. doi: 10.1371/journal.pcbi.1007069 (PMC6555534; doi:10.1371/journal.pcbi.1007069)

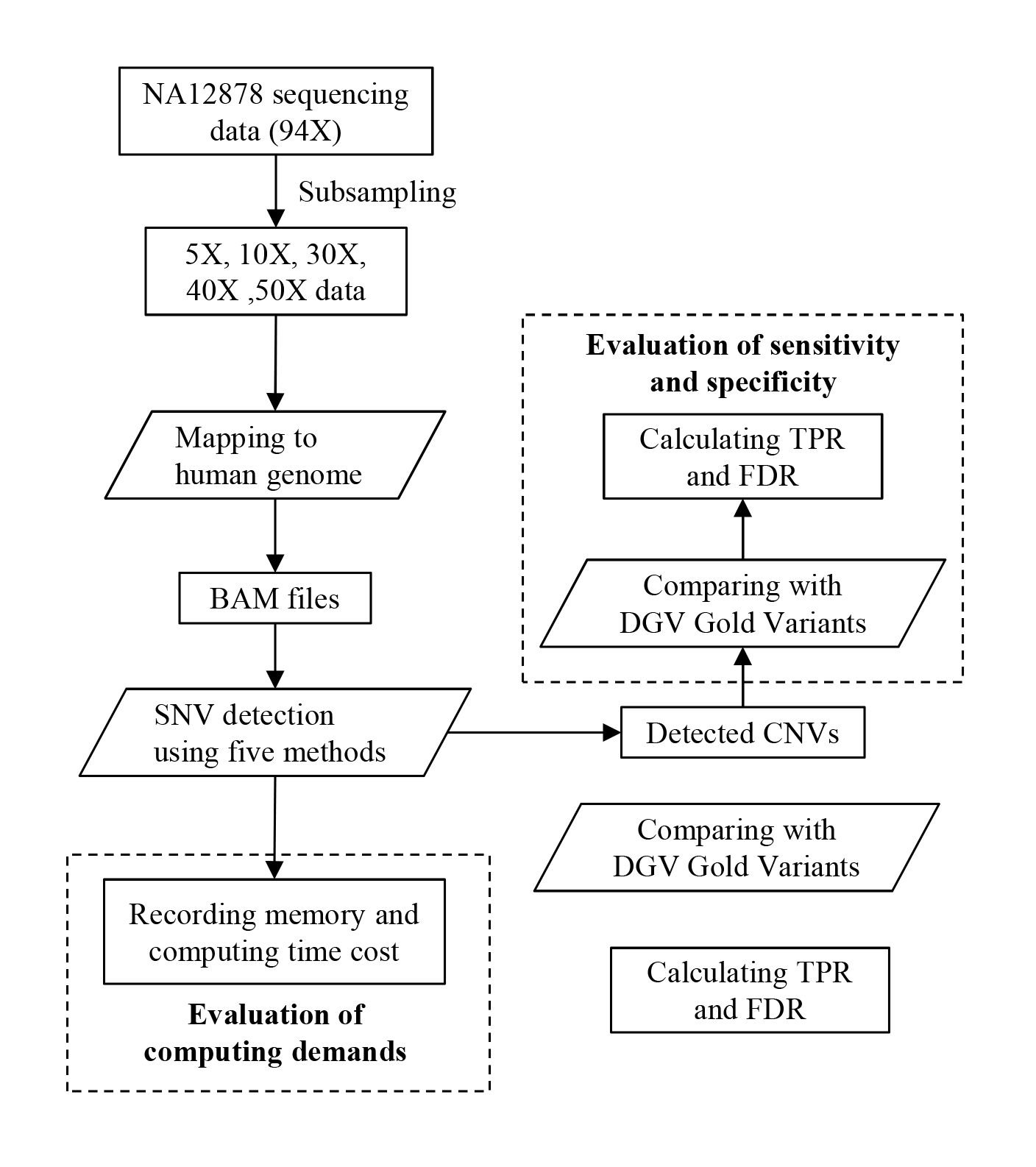

Supplement: S1 Fig — (TIF) [file pcbi.1007069.s001.tif]

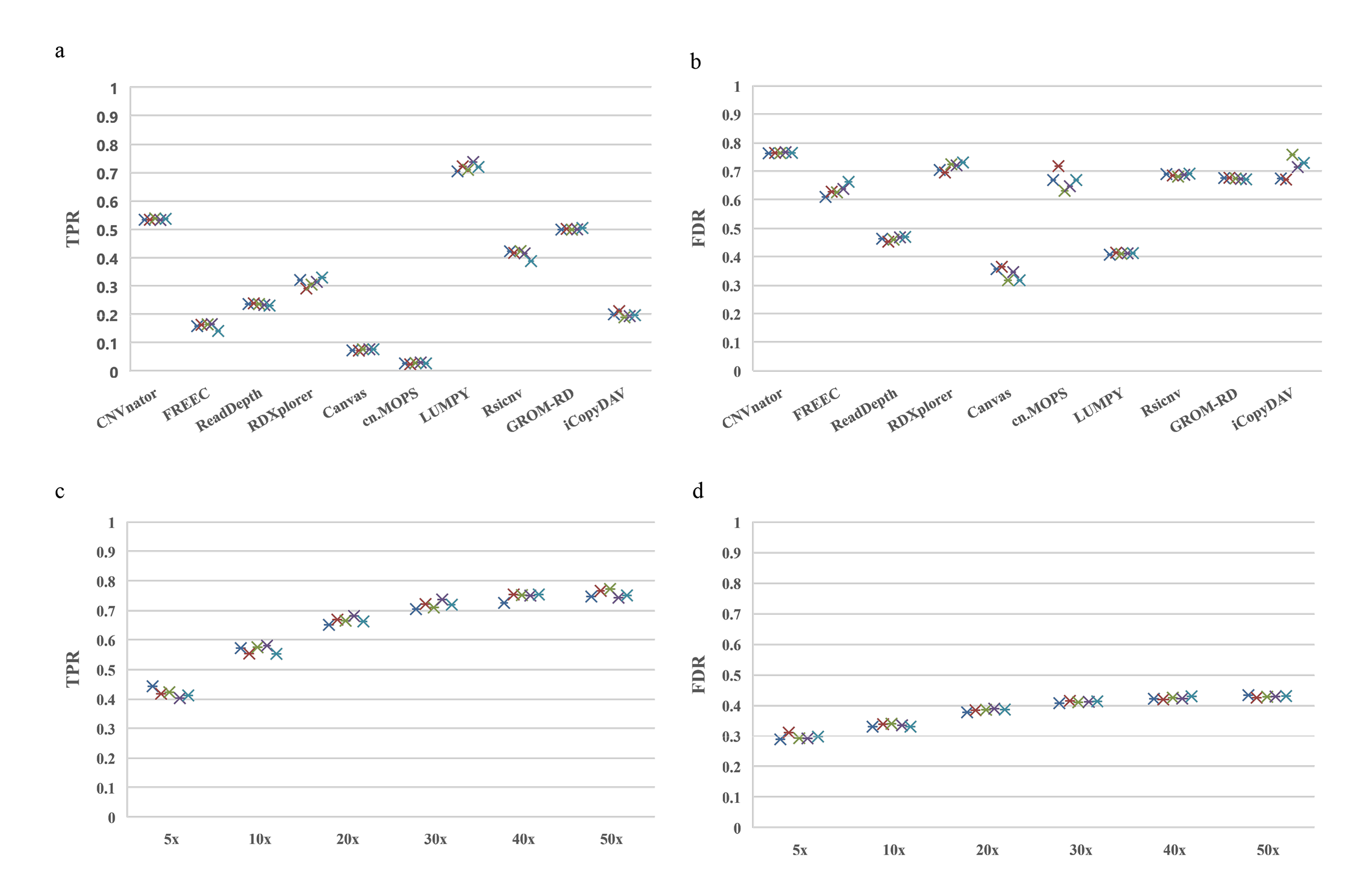

Supplement: S2 Fig — (a) TPR of the ten application at 30X depth using five times subsampling. (b) FDR of the ten application at 30X depth using five times subsampling. (c) TPR of Lumpy from 5X to 50X depth using five times subsampling at each depth. (d) FDR of Lumpy from 5X to 50X depth using five times subsampling at each depth. (TIF) [file pcbi.1007069.s002.tif]
