# Supplementary material for: Comprehensively benchmarking applications for detecting copy number variation
Source: PLoS Comput Biol. 2019 May 28;15(5):e1007069. doi: 10.1371/journal.pcbi.1007069 (PMC6555534; doi:10.1371/journal.pcbi.1007069)
Supplement: S1 Table — (DOCX) [file pcbi.1007069.s003.docx]

**S1 Table. Detailed information of the tested software**

| **Software** | **Methods** | **Features** | **Parameter** |
| --- | --- | --- | --- |
| Canvas | RD | A favorable tool for both somatic and germline CNV detection in large-scale sequencing studies, which imple-ments all steps of the variant calling workflow | Germline-WGS |
| cn.MOPS | RD | Modelling of read depths across samples at each genomic position using mixture Poisson model | singlecn.mops |
| CNVnator | RD | Using mean-shift approach and performing multiple-bandwidth partitioning and GC correction | -his 100  -stat 100  -partition 100  -call 100 |
| Control-FREEC | RD | Correcting copy number using matched case-control samples or GC contents | window = 500  step=100  ploidy = 2  sex=XX or XY  readCountThreshold=10  breakPointThreshold=0.8 |
| GROM-RD | RD | A control-free CNV algorithm combining excessive coverage masking, GC bias mean and variance normalization | default |
| iCopyDAV | RD | A modular-framework based on DoC approaches | --win 100  -mapThres 0.6 |
| LUMPY | RD, PEM | Integrates the CNV detection methods of RD and PEM, and allows for more sensitive CNV discovery | window=500bp step=100bp ploidy=2 |
| RDXplorer | RD | Detecting CNVs through event-wise testing algorithm on normalized read depth of coverage | winSize=100  baseCopy=2 |
| ReadDepth | RD | Using breakpoints to increase the resolution of CNV detection from low-coverage reads | readLength=100  fdr=0.01  overDispersion =3  gcWindowSize =100  percCNGain=0.05  percCNLoss=0.05  chunkSize=5e6  maxCores=8  readCores=8 |
| RSICNV | RD | Using the robust segment identification algorithm with negative binomial transformations | -m 400 |
